# Genotype-Phenotype Correlations and Characterization of Medication Use in Inherited Myotonic Disorders

**DOI:** 10.3389/fneur.2020.00593

**Published:** 2020-06-26

**Authors:** Alayne P. Meyer, Jennifer Roggenbuck, Samantha LoRusso, John Kissel, Rachel M. Smith, David Kline, W. David Arnold

**Affiliations:** ^1^Division of Human Genetics, The Ohio State University, Columbus, OH, United States; ^2^Department of Neurology, The Ohio State University, Columbus, OH, United States; ^3^Department of Biomedical Informatics, Center for Biostatistics, The Ohio State University, Columbus, OH, United States

**Keywords:** myotonia, channelopathies, inherited, treatment, genotype-phenotype, myotonic dystrophy, myotonia congenita, paramyotonia congenital

## Abstract

**Introduction:** Inherited myotonic disorders are genetically heterogeneous and associated with overlapping clinical features of muscle stiffness, weakness, and pain. Data on genotype-phenotype correlations are limited. In this study, clinical features and treatment patterns in genetically characterized myotonic disorders were compared.

**Methods:** A retrospective chart review was completed in patients with genetic variants in *CLCN1, SCN4A, DMPK*, and *CNBP* to document clinical signs and symptoms, clinical testing, and antimyotonia medication use.

**Results:** A total of 142 patients (27 *CLCN1*, 15 *SCN4A*, 89 *DMPK*, and 11 *CNBP*) were reviewed. The frequency of reported symptoms (stiffness, weakness, and pain) and electromyographic spontaneous activity were remarkably similar across genotypes. Most patients were not treated with antimyotonia agents, but those with non-dystrophic disorders were more likely to be on a treatment.

**Discussion:** Among the features reviewed, we did not identify clinical or electrophysiological differences to distinguish *CLCN1*- and *SCN4A*-related myotonia. Weakness and pain were more prevalent in non-dystrophic disorders than previously identified. In addition, our results suggest that medical treatments in myotonic disorders may be under-utilized.

## Introduction

Myotonia is a phenomenon of skeletal muscle hyperexcitability that impairs muscle relaxation following contraction or percussion (1–4). Myotonia can be clinically evident with visually appreciable delays in muscle relaxation, but can also be subclinical with myotonic discharges noted on electromyography (EMG) in the absence of overt clinical signs. Etiologies of myotonia include monogenic inherited causes as well as acquired causes such as hypothyroidism, denervation, inflammatory myopathies, or toxic myopathies (5). Inherited myotonic disorders are generally divided into two major categories, dystrophic and non-dystrophic, with the major differentiating factor being the presence of progressive muscle degeneration in patients with dystrophic forms of myotonia (1, 3, 6, 7). Dystrophic myotonic disorders include myotonic dystrophy type I (DM1), characterized by prominent distal limb weakness and multisystem disease, and myotonic dystrophy type II (DM2, also known as proximal myopathic myotonia or PROMM), characterized by proximal limb weakness with less prevalent multisystem disease (3).

DM1 is caused by a pathogenic CTG expansion in *DMPK* (>50 repeats) which results in altered splicing of *CLCN1*, as well as other genes, leading to reduced chloride conductance and myotonia (8–10). Intergenerational expansion of repeat size may be observed, with an increased repeat size correlating with earlier age of onset and increased severity of symptoms, a phenomenon known as anticipation (9–11). The most severe form of DM1 is seen in individuals with congenital symptoms, typically caused by a repeat size of >1000 (10, 11). DM2 is caused by a pathogenic CCTG expansion in *CNBP* and also affects splicing of *CLCN1*. Genetic anticipation and congenital onset of disease are not observed in DM2 (9–13). Typically, DM2 is milder and has a later age of onset than DM1. Additionally, repeat length is not correlated with severity of symptoms or age of onset (9–12). Pain is a more prominent feature in many individuals with DM2 and may be the presenting symptom, often leading to misdiagnoses (13). Patients with DM1 and DM2 also experience systemic complications including progressive cardiac conduction defects, respiratory insufficiency, premature cataracts, daytime hypersomnolence, gastrointestinal and endocrine dysfunction, cognitive and behavioral deficits, and increased malignancy rates (9–11).

The non-dystrophic myotonias are caused by mutations of specific skeletal muscle ion channels and are usually categorized on the basis of the ion channel affected, inheritance pattern, and clinical features. The two skeletal muscle ion channels that are associated with non-dystrophic disorders include chloride (*CLCN1)* and sodium (*SCN4A)* channels. The non-dystrophic myotonias are known to be highly variable in expression, leading to missed or delayed diagnosis in many cases (1, 14).

Dominant and recessive pathogenic variants in *CLCN1* cause myotonia congenita (MC), the most common inherited muscle channelopathy (6, 7). Variants lead to loss-of-function and dominant negative effects in the CLC-1 channel causing reduced chloride conductance and membrane hyperexcitability (2–4). Both forms are typically characterized by childhood onset muscle stiffness (1, 3). Stiffness can be triggered by emotional surprise and aggravated by cold temperatures and pregnancy. Repetitive motion or “warm up” can alleviate these symptoms (1, 2, 6, 7, 14). Recessively inherited MC often causes more severe symptoms and may cause a slowly progressive muscle weakness that may be identified on clinical examination, as well as transient weakness (1, 3, 14).

Paramyotonia congenita (PMC), sodium channel myotonia (SCM), and hyperkalemic periodic paralysis (HyperPP) are all caused by dominant gain-of-function variants in *SCN4A* (15). These variants lead to an excessive inward sodium ion current which causes muscle hyperexcitability, leading to myotonia, or transient loss of excitability, causing periodic paralysis (16). Symptoms of PMC typically begin in the first decade of life and include myotonia that is induced by cold or repeated muscle activity (paradoxical myotonia or paramyotonia), as well as episodic weakness triggered by exercise, cold, potassium ingestion, or fasting (6, 7, 12, 15, 16). On clinical examination patients with variants in *SCN4A* often display eyelid myotonia that worsens with repetitive eyelid closure (1). SCM is characterized by pure, often painful, myotonia without episodic weakness and typically not triggered by cold temperatures (1, 6, 7, 15). HyperPP is characterized by recurrent episodes of weakness triggered by exercise, potassium ingestion, or emotional stress that can last hours to days. Over time, a slowly progressive permanent weakness may occur in HyperPP (16). Hypokalemic periodic paralysis (HypoPP) is also caused by mutations in *SCN4A* and is usually not associated with myotonia, although rarely is has been described in patients with homozygous loss-of-function variants (17).

Treatment of myotonic disorders is generally influenced by symptom severity and ability to control symptoms through avoidance of triggers (6). Pharmacological treatment may not be necessary in all patients, but patients who accept medical treatment may experience significant improvement in myotonia symptoms, including pain (5). There are currently no FDA approved medications for the treatment of myotonia; however, mexiletine has been approved in the EU as an antimyotonia agent. A multinational study has found that only 40% of patients received treatment for this symptom (14). Off-label antimyotonia treatments include anti-arrhythmic, anti-epileptic, and anti-depressant medications, which have shown clinical benefit, usually in small case series or single case reports. Mexiletine has demonstrated efficacy in multiple controlled studies in non-dystrophic myotonia and in DM1 (18, 19). In addition, a recent n-of-1 aggregate study also showed efficacy in a study cohort of 27 patients with non-dystrophic myotonia (20). Similarly, lamotrigine has been demonstrated to be effective in non-dystrophic myotonia (21). There is a lack of published data detailing usage and efficacy of antimyotonia agents in clinical practice.

In this study, we retrospectively reviewed patient-reported symptoms and clinical data of patients with genetically defined myotonic disorders seen at a large tertiary center. We aimed to characterize the phenotypic profiles of each disorder by comparing symptom profiles between the disorders as well as the usage of commonly prescribed antimyotonia agents.

## Materials and Methods

A retrospective chart review was performed of patients with inherited myotonic disorders seen at The Ohio State University Wexner Medical Center from March 2009 to December 2018. This study and waiver of consent was approved by the Ohio State University institutional review board.

### Subjects

Initial patient search was conducted to identify all patients with myotonia-related diagnostic codes. A complete list of codes utilized are found in Table 1. Patients were included in the review if they or a family member had a documented variant in *CLCN1* or *SCN4A* or a pathogenic expansion in *DMPK* or *CNBP*. Patients were excluded if they had absence of myotonia both clinically and electrically or if they had a pathogenic or likely pathogenic variant identified in a second gene related to neuromuscular disease.

**Table 1:** List of diagnostic codes utilized in the search for candidates meeting inclusion criteria.

**ICD-9/ICD-10**	**Diagnosis**
359.39/G71.19	Myotonia fluctuans
359.21/G71.11	Myotonia atrophica
359.23/G71.13	Myotonia chondrodystrophica
359.22/G71.12	Myotonia congenita
728.85, 319, 756.50/M62.89, F79, Q78.9	Myotonia with intellectual disability and skeletal anomaly
359.24, E980.5/G71.14	Myotonia, drug-induced
359.3/G72.3	Periodic myotonia
794.17/R94.131	Myotonic changes present on EMG
V83.89/Z14.8	Carrier of myotonic dystrophy
271/E74.02	Pompe disease
796.4/R89.0	Low acid maltase in muscle determined by biopsy
792.9/R89.0	Low acid maltase levels in fibroblasts
359.0, V84.89/G71.2, Z15.89	Autosomal dominant centronuclear myopathy associated with mutation in DMN2 gene
359.89, 359.0/G72.89, G71.2	Myofibrillar myopathy

### Chart Review

Data were collected on patient demographics, patient-reported symptoms (including stiffness, weakness, pain, cramping, and exacerbating factors), family history, and medication history. Physical examination data were documented, including presence of clinical myotonia and weakness, creatine kinase levels, and EMG data. Presence of periodic paralysis and paradoxical myotonia were not ascertained. Genetic testing results, including genetic tests completed and complete variant data, including pathogenic classification (pathogenic, likely pathogenic, or uncertain), were ascertained via review of laboratory reports. For patient-reported symptoms and clinical examination data, each symptom was recorded as present only if the chart note specifically stated that the patient had that symptom. To avoid ascertainment bias, if a symptom was not recorded as present or absent in the chart note, it was listed as “unknown” and data on that symptom in that patient was not utilized in the statistical analysis. Exacerbating factors were recorded as present if documented in the patient's chart, and recorded as absent if it was not documented or if specifically noted as not being present. Muscle weakness was considered present on clinical examination if a patient scored a “4” or less in any muscle group at any clinical visit during manual muscle testing by a neuromuscular specialist. If multiple creatine kinase levels were available, the highest value was recorded. Reference ranges used were 30-220 U/L for men and 30-184 U/L for women. Values recorded from electromyography (EMG) studies included number of muscles tested and number of muscles with abnormal spontaneous activity, including myotonia, positive sharp waves, and fibrillation potentials. If multiple EMG studies were performed, the number of muscles tested and the number of muscles with abnormal spontaneous activity were summed across all studies for each patient in each of these categories. Usage of the following medications was recorded: Acetazolamide, Clomipramine, Diazapam, Dichlorphenamide, Dispyramide, Imipramine, Lamotrigine, Mexiletine, Nifedipine, Phenytoin, Procainamide, Quinine, Ranolazine, Taurine, Thiazides, and Tocainide.

### Statistical Analysis

Descriptive statistics were utilized to create the phenotypic profile for each genotype. Mean values were utilized for participant age and age of symptom onset. For the remaining chart review data, percentages were utilized to depict the incidence of a given phenotypic characteristic by genotype. Any characteristic that was not explicitly recorded as present or absent was not utilized in the statistical analysis for that characteristic. Comparison of symptoms across genotype groups and dystrophic versus non-dystrophic groups were made utilizing a Fisher's exact test. Descriptive statistics were utilized to determine the proportion of each genotype group who had trialed and were currently taking an antimyotonia agent. Patients currently taking a medication at the time of review were divided by genotype and type of medication utilized.

## Results

### Demographics

**Figure 1:**
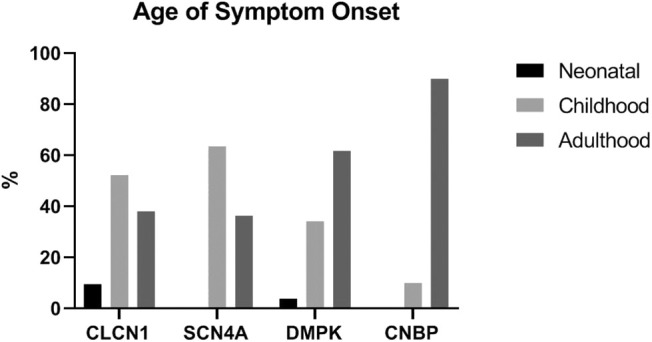
Age range at time of symptom onset by genotype. Neonatal <1 year old, childhood 1−18 years old, adulthood >18 years old.

A total of 142 patients were included in this study: 27 had one or more variants in *CLCN1*, 15 had a variant in *SCN4A*, 89 had an expansion in *DMPK*, and 11 had an expansion in *CNBP*. The average age of symptom onset for individuals with *CLCN1, SCN4A, DMPK*, and *CNBP* variants was 16.5 years [standard deviation (*SD*) 12.1], 23.6 years (*SD* 21.0), 28.0 years (*SD* 15.9), and 40.7 (*SD* 12.0), respectively. Figure 1 depicts symptom onset by age category (neonatal being defined as <1 year of age, childhood as 1–18 years old, and adulthood being greater than 18 years old) and genotype. Most participants were Caucasian (83.8%) and female (81.5% *CLCN1*, 73.3% *SCN4A*, 60.7% *DMPK*, 45.5% *CNBP*). A total of 13 individuals included in the study were deceased at the time of chart review. Eleven of these individuals had *DMPK* expansions, with the average age of death among these individuals being 54.3 years (*SD* 10.4). The other two deaths both occurred at the age of 68, one in an individual with an expansion in *CNBP* and the other in an individual with a variant in *CLCN1*.

### Genetic Variants

Of the 27 individuals with at least one variant in *CLCN1*, 23 individuals had one variant identified and 4 had two variants with apparent autosomal recessive inheritance of disease. A total of 13 different variants were identified in the *CLCN1* cohort with eight being classified as pathogenic, two as likely pathogenic, and three as variants of uncertain significance (VUS). No individuals with an *SCN4A* variant were identified to have more than one variant in this gene on their genetic lab report. In the *SCN4A* cohort, 9 different variants were identified with eight being classified as pathogenic and one as a VUS. A full list of genotypes identified and corresponding myotonia phenotypes are summarized in Tables 2–4. Additional details on the genetic testing performed for patients with unclear results (including those with single variants previously associated with recessive disease, those with variants of uncertain significance and those possessing variants with conflicting interpretations) are summarized in Supplementary Table 1. Expansion length in *DMPK* ranged from 74 repeats to 2,450 repeats. Among individuals in this cohort, 5.6% (*n* = 5) were identified as having <100 repeats, 67.4% (*n* = 60) were identified as having between 100 and 1000 repeats, and 27% (*n* = 24) were identified as having greater than 1,000 repeats in at least part of the sample (in the case of mosaicism). Due to the retrospective nature of this study, we were unable to ascertain the subtype of DM1 for each patient; however, only two patients had onset of symptoms in infancy, while the remaining had childhood or adult onset symptoms.

**Table 2:** List of patients with one variant identified in *CLCN1*.

**c**.	**p**.	**Lab reported classification**	**ClinVar classification**	**Apparent inheritance pattern**	**Clinical myotonia**	**Spontaneous activity on EMG**
c.469delC	p.Leu157Phefs*13	Pathogenic	Pathogenic	Negative family Hx	No	Yes (8/11)
c.501C>G	p.Phe167Leu	VUS	Conflicting	Negative family Hx	Yes	Yes (4/10)
c.592C>G	p.Leu198Val	VUS	Conflicting	AD	Yes	Yes (3/5)
c.689G>A	p.Gly230Glu	Pathogenic	Pathogenic	AD	Yes	Yes (3/3)
c.689G>A	p.Gly230Glu	Pathogenic	Pathogenic	AD	Yes	Unknown
c.689G>A	p.Gly230Glu	Pathogenic	Pathogenic	AD	Unknown	Yes (3/9)
c.689G>A	p.Gly230Glu	Pathogenic	Pathogenic	AD	Yes	Unknown
c.689G>A	p.Gly230Glu	Pathogenic	Pathogenic	AD	No	Unknown
c.689G>A	p.Gly230Glu	Pathogenic	Pathogenic	AD	Yes	Yes (3/3)
c.689G>A	p.Gly230Glu	Pathogenic	Pathogenic	AD	Yes	Unknown
c.689G>A	p.Gly230Glu	Pathogenic	Pathogenic	Unknown	Unknown	Unknown
c.689G>A	p.Gly230Glu	Pathogenic	Pathogenic	Unclear	Yes	Unknown
c.929C>T	p.Thr310Met	Pathogenic	Pathogenic	AD	No	Unknown
c.929C>T	p.Thr310Met	Pathogenic	Pathogenic	AD	Unknown	Unknown
c.937G>A	p.Ala313Thr	Pathogenic	Pathogenic	AD	Unknown	Yes (8/17)
c.937G>A	p.Ala313Thr	Pathogenic	Pathogenic	AD	Yes	Yes
c.1167-10 T>C	Intronic	Likely pathogenic	Likely pathogenic	Unclear	Yes	Yes (8/8)
c.1444G>C	p.Gly482Arg	Likely pathogenic	Likely pathogenic	AD	No	Yes (10/10)
c.1655A>G	p.Gln552Arg	Pathogenic	Conflicting (LP/P)	AD	Yes	Yes
c.2680C>T	p.Arg894Ter	Pathogenic	Conflicting (LP/P)	Unknown	No	Unknown
c.2680C>T	p.Arg894Ter	Pathogenic	Conflicting (LP/P)	Unknown	Yes	Unknown
c.2848G>A	p.Glu950Lys	VUS	N/a	AD	Yes	Yes (3/5)

*For each patient, the classification of the variant as listed on the original laboratory report as well as the classification in the ClinVar database are included. Apparent inheritance is based on family history information identified in the chart. Patients with family members with a diagnosis of myotonia congenita (with or without genetic confirmation), EMG positive for myotonia or reported symptoms of myotonia or stiffness were categorized as autosomal dominant inheritance (“AD”). Patients with family history information available which was negative for any of these symptoms were categorized as “negative family hx.” Patients with no family history information listed in the chart were categorized as “unknown” and patients with family history of symptoms of unclear diagnostic significance (pain, cramping or weakness) were categorized as “unclear.” Presence of clinical myotonia is listed as positive, negative or unknown for each patient. Spontaneous activity on EMG is listed as positive, negative or unknown. In parentheses, the number of muscles affected with spontaneous activity divided by the total muscles tested on EMG for that individual is recorded (if known)*.

**Table 3:** List of patients with two variants identified in *CLCN1*.

**c**.	**p**.	**Lab reported classification**	**ClinVar classification**	**Apparent inheritance pattern**	**Clinical myotonia**	**Spontaneous activity on EMG**
c.689G>A	p.Gly230Glu	Pathogenic;	Pathogenic;	AD	Yes	Unknown
c.1444G>C	p.Gly482Arg	Likely pathogenic	Likely pathogenic			
c.568G>A	p.Gly190Arg	VUS;	Conflicting;	Negative family Hx	Unknown	Yes
c.1238T>G	p.Phe413Cys	Pathogenic	Pathogenic			
c.979G>A	p.Val327Ile	Pathogenic;	Pathogenic;	Negative family Hx	Yes	Unknown
c.1262G>T	p.Arg421Leu	VUS	Likely pathogenic			
c.1238T>G	p.Phe413Cys	Pathogenic;	Pathogenic;	Negative family Hx	Yes	Yes (3/5)
c.2680C>T	p.Arg894Ter	Pathogenic	Conflicting (LP/P)			
c.409T>G	p.Tyr137Asp	Likely pathogenic;	Likely pathogenic;	Negative family Hx	Yes	Yes (18/18)
c.1238T>G	p.Phe413Cys	Pathogenic	Pathogenic			

*For each patient, the classification of the variant as listed on the original laboratory report as well as the classification in the ClinVar database are included. Apparent inheritance is based on family history information identified in the chart. Patients with family members with a diagnosis of myotonia congenita (with or without genetic confirmation), EMG positive for myotonia or reported symptoms of myotonia or stiffness were categorized as autosomal dominant inheritance (“AD”). Patients with family history information available which was negative for any of these symptoms were categorized as “negative family hx.” Patients with no family history information listed in the chart were categorized as “unknown” and patients with family history of symptoms of unclear diagnostic significance (pain, cramping or weakness) were categorized as “unclear.” Presence of clinical myotonia is listed as positive, negative or unknown for each patient. Spontaneous activity on EMG is listed as positive, negative or unknown. In parentheses, the number of muscles affected with spontaneous activity divided by the total muscles tested on EMG for that individual is recorded (if known)*.

**Table 4:** List of patients with variants in *SCN4A*.

**c**.	**p**.	**Lab reported classification**	**ClinVar classification**	**Apparent inheritance pattern**	**Clinical myotonia**	**Spontaneous activity on EMG**
c.1333G>A	p.Val445Met	Pathogenic	Pathogenic	AD	Yes	Yes (6/6)
c.1333G>A	p.Val445Met	Pathogenic	Pathogenic	AD	Yes	Yes (2/2)
c.1333G>A	p.Val445Met	Pathogenic	Pathogenic	AD	No	Yes (3/3)
c.2078T>C	p.Ile693Thr	Pathogenic	Pathogenic	AD	Yes	Unknown
c.3917G>C	p.Gly1306Ala	Pathogenic	Pathogenic	Unclear	No	Unknown
c.3917G>C	p.Gly1306Ala	Pathogenic	Pathogenic	Unknown	Yes	Unknown
c.3917G>C	p.Gly1306Ala	Pathogenic	Pathogenic	AD	Yes	Unknown
c.3938C>T	p.Thr1313Met	Pathogenic	Pathogenic	Negative Family Hx	Yes	Yes (7/7)
c.4343G>A	p.Arg1448His	Pathogenic	Pathogenic	AD	Yes	Yes (5/5)
c.4343G>A	p.Arg1448His	Pathogenic	Pathogenic	Unknown	No	Unknown
c.4343G>A	p.Arg1448His	Pathogenic	Pathogenic	AD	Yes	Yes (2/2)
c.4372G>T	p.Val1458Phe	Likely Pathogenic	VUS	Negative Family Hx	Unknown	Yes (16/22)
c.4386C>G	p.Ile1462Met	Pathogenic	VUS	Unknown	Unknown	Yes
c.4765G>A	p.Val1589Met	Pathogenic	Pathogenic	AD	Yes	Yes (2/2)
c.5126A>G	p.Asn1709Ser	VUS	VUS	Unclear	Unknown	Yes (2/4)

*For each patient, the classification of the variant as listed on the original laboratory report as well as the classification in the ClinVar database are included. Apparent inheritance is based on family history information identified in the chart. Patients with family members with a diagnosis of paramyotonia congenita or sodium channel myotonia (with or without genetic confirmation), EMG positive for myotonia or reported symptoms of myotonia or stiffness were categorized as autosomal dominant inheritance (“AD”). Patients with family history information available which was negative for any of these symptoms were categorized as “negative family hx.” Patients with no family history information listed in the chart were categorized as “unknown” and patients with family history of symptoms of unclear diagnostic significance (pain, cramping or weakness) were categorized as “unclear.” Presence of clinical myotonia is listed as positive, negative or unknown for each patient. Spontaneous activity on EMG is listed as positive, negative or unknown. In parentheses, the number of muscles affected with spontaneous activity divided by the total muscles tested on EMG for that individual is recorded (if known)*.

### Patient Reported Symptoms

Stiffness was reported by all individuals in our *CLCN1* cohort and was reported in ~80% of the remaining three groups. Weakness was reported in a similar proportion of individuals with non-dystrophic myotonias, 65.2% (*n* = 15) of individuals with *CLCN1* variants, and 69.2% (*n* = 9) *SCN4A* variants. Weakness was more often reported in individuals with dystrophic myotonia than non-dystrophic myotonia, with 90.8% (*n* = 79) of individuals with *DMPK* expansions and 90% (*n* = 9) with *CNBP* expansions reporting this as a symptom. History of pain was reported by all individuals with *CNBP* expansions (*n* = 11), while it was found in 50–70% of the remaining three groups. Presence of muscle cramping was similar across all groups with 50% (*n* = 6) of individuals with *SCN4A* variants and 30–40% of the remaining three groups reporting this. Cold as an exacerbating factor for symptoms was most commonly reported in individuals with non-dystrophic myotonias, with 40.7% (*n* = 11) of individuals with *CLCN1* variants and 46.7% (*n* = 7) of individuals with *SCN4A* variants reporting this compared to 36.4% (*n* = 4) of individuals with *CNBP* expansions and in 7.9% (*n* = 7) of individuals with *DMPK* expansions. A summary of patient reported symptom data is in Table 5.

**Table 5:** Summary of patient reported symptoms by genotype.

**Symptom**		**Non-dystrophic**		**Dystrophic**		**Total**	***p*-value**
		**CLCN1**	**SCN4A**	**DMPK**	**CNBP**		
Stiffness	Yes	24 (100.0%)	11 (78.6%)	62 (78.5%)	8 (80.0%)	105 (82.7%)	0.0371
	Unknown	3	1	10	1	15	
Weakness	Yes	15 (65.2%)	9 (69.2%)	79 (90.8%)	9 (90.0%)	112 (84.2%)	0.0072
	Unknown	4	2	2	1	9	
Pain	Yes	18 (69.2%)	8 (53.3%)	50 (59.5%)	10 (100.0%)	86 (63.7%)	0.0417
	Unknown	1	0	5	1	7	
Cramping	Yes	8 (34.8%)	6 (50.0%)	30 (37.5%)	3 (30.0%)	47 (37.6%)	0.8021
	Unknown	4	3	9	1	17	
Cold exacerbation	Yes	11 (40.7%)	7 (46.7%)	7 (7.9%)	4 (36.4%)	29 (20.4%)	<0.0001

*P-values were obtained using Fisher's exact test*.

### Clinical Examination

Most individuals in the overall cohort had at least one form of clinical myotonia with 69–94% of each group having this present on physical examination. Hand grip myotonia was least common in individuals with expansions in *CNBP* at 16.7% (*n* = 1) while both hand grip and percussion myotonia were most commonly identified in individuals with *DMPK* expansions at 85.1% (*n* = 63) and 94.6% (*n* = 70), respectively. Muscle weakness was most common in the individuals with *DMPK* expansions at 91.8% (*n* = 78) followed by individuals with *CNBP* expansions at 54.5% (*n* = 6). However, 16% (*n* = 4) of individuals with *CLCN1* variants and 26.7% (*n* = 4) of individuals with *SCN4A* variants were also found to have weakness in at least one muscle group. Distribution of weakness on MMT was most commonly identified as proximal only in *SCN4A* patients, was evenly split between proximal only and both proximal and distal in *CLCN1* and *CNBP* patients and was most commonly identified as both proximal and distal in patients with expansions in *DMPK*. A summary of clinical examination data is in Table 6.

**Table 6:** Summary of clinical examination findings by genotype.

**Symptom**		**Non-dystrophic**		**Dystrophic**		**Total**	***p*-value**
		**CLCN1**	**SCN4A**	**DMPK**	**CNBP**		
Clinical myotonia	Yes	17 (73.9%)	9 (69.2%)	75 (93.8%)	7 (77.8%)	108 (86.4%)	0.0067
	Unknown	4	2	9	2	17	
Hand grip myotonia	Yes	14 (70.0%)	9 (75.0%)	63 (85.1%)	1 (16.7%)	87 (77.7%)	0.0022
	Unknown	7	3	15	5	30	
Percussion myotonia	Yes	12 (70.6%)	4 (57.1%)	70 (94.6%)	5 (62.5%)	91 (85.8%)	0.0006
	Unknown	10	8	15	3	36	
Muscle weakness (on MMT)	Yes	4 (16.0%)	4 (26.7%)	78 (91.8%)	6 (54.5%)	92 (67.6%)	<0.0001
	Unknown	2	0	4	0	6	

*P-values were obtained using Fisher's exact test*.

### EMG Results

**Figure 2:**
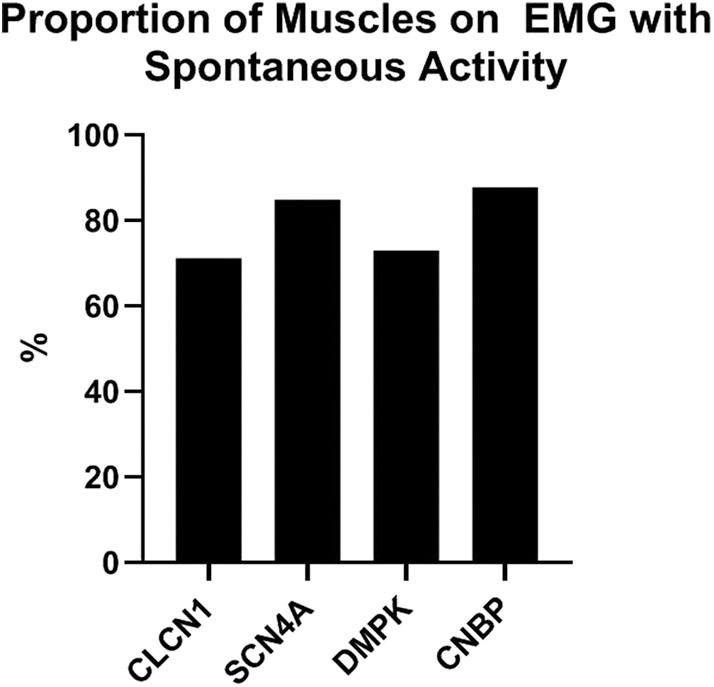
Proportion of muscles on EMG with abnormal spontaneous activity by genotype.

In this cohort, individuals with *DMPK* expansions were least likely to have had an EMG performed at 43.8% of the group compared to 70–100% of the remaining three groups. The proportion of muscles with spontaneous activity was similar among the four groups ranging from 71 to 88%. Proportion of muscles with spontaneous activity by genotype is depicted in Figure 2.

### Creatine Kinase Levels

CK levels were available for 81 participants. Values were similar for the non-dystrophic groups, with the average being 157.8 U/L (*SD* 153.6) for individuals with *CLCN1* variants and 168.6 U/L (*SD* 137.8) for individuals with *SCN4A* variants. Values were higher for the individuals with *DMPK* and *CNBP* expansions with the averages being 243.4 and 345.6 U/L, respectively. The proportion of individuals with an abnormal CK level was 23.1% (*n* = 3) of individuals with *CLCN1* variants, 37.5% (*n* = 3) of individuals with *SCN4A* variants, 54% (*n* = 27) of individuals with *DMPK* expansions, and 40% (*n* = 4) of individuals with *CNBP* expansions.

### Medication Usage

**Figure 3:**
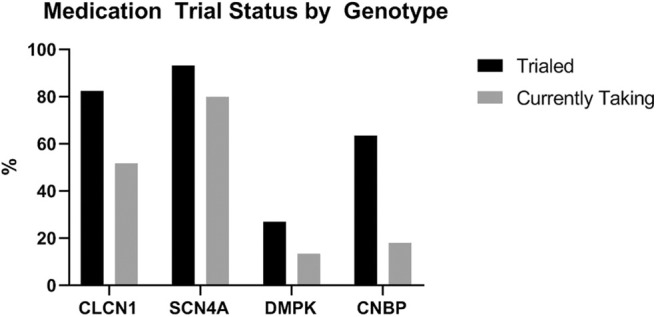
Proportion of individuals, by genotype, who had ever trialed an antimyotonia medication and who were currently taking one at the time of chart review.

**Figure 4:**
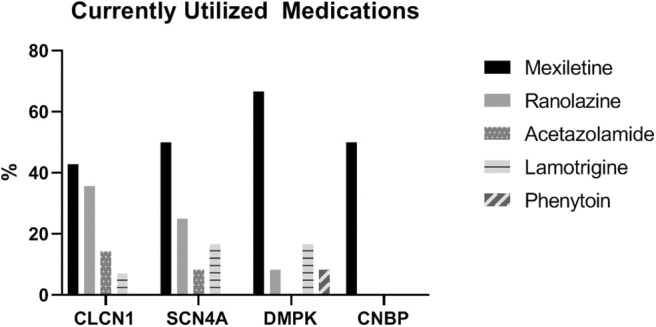
Proportion (of those currently taking a medication) of individuals taking each type of antimyotonia agent.

Patients with *DMPK* expansions were the least likely to have trialed an antimyotonia medication at 27% (*n* = 24). Medications had been trialed in 85.2% of individuals with *CLCN1* variants (*n* = 23), 93.3% of individuals with *SCN4A* variants (*n* = 14), and 63.6% of individuals with *CNBP* expansions (*n* = 7). Genotype specific medication trialing data is summarized in Figure 3. Of those who trialed medications, 56.5% (*n* = 13) of *CLCN1* patients, 66.7% (*n* = 10) of *SCN4A* patients, and 8.3% (*n* = 2) of *DMPK* patients had tried more than one medication for myotonia. No patients with *CNBP* expansion had trialed multiple medications. The most commonly trialed medication across all four groups was mexiletine at 40.4% of individuals who trialed any medication (*n* = 42). Currently utilized medications by genotype are depicted by Figure 4. More individuals with non-dystrophic myotonias were currently taking at least one medication for myotonia with 51.9% (*n* =14) of individuals with *CLCN1* variants and 80.0% (*n* = 12) of individuals with *SCN4A* variants compared to 13.5% (*n* = 12) of individuals with *DMPK* expansions, and 18.2% (*n* = 2) of individuals with *CNBP* expansions. Out of the patients trialing at least one medication, none with *SCN4A* variants had discontinued all medication use for myotonia. Patients with dystrophic myotonias more commonly had discontinued all antimyotonia agents with 50.0% (*n* = 12) patients with *DMPK* expansions and 71.4% (*n* = 5) patients with *CNBP* expansions having done so compared to 17.4% (*n* = 4) of individuals with *CLCN1* variants. Reason for discontinuation was given for 30 (60%) of the medications stopped. The frequency that each medication was stopped due to cost, drug interactions, efficacy, or side effects is presented recorded in Table 7. The most common reasons for discontinuation overall were lack of efficacy (32%, *n* = 16) and side effects (20%, *n* = 10). For mexiletine, reason for discontinuation was known in nine of cases with 21.2% (*n* = 4) being due to lack of efficacy and 26.3% (*n* = 5) due to side effects.

**Table 7:** Summary of reasons patients discontinued commonly utilized antimyotonia agents.

**Reason**	**Procainamide**	**Phenytoin**	**Quinine**	**Mexiletine**	**Acetazolamide**	**Ranolazine**	**Lamotrigine**	**Total**
Cost	–	–	1 (33.3%)	–	–	2 (16.7%)	–	3 (6.0%)
Drug interactions	–	–	–	–	–	1 (8.3%)	–	1 (2.0%)
Efficacy	1 (100.0%)	3 (42.9%)	–	4 (21.1%)	4 (80.0%)	4 (33.4%)	–	16 (32.0%)
Side effects	–	1 (14.3%)	–	5 (26.3%)	1 (20.0%)	2 (16.7%)	1 (50.0%)	10 (20.0%)

*No patients discontinued medications due to allergic reaction.

**Not all fields will add to 100% as not all individuals discontinuing a medication had a reason in their chart.

### Dystrophic (D) vs. Non-dystrophic (ND)

**Figure 5:**
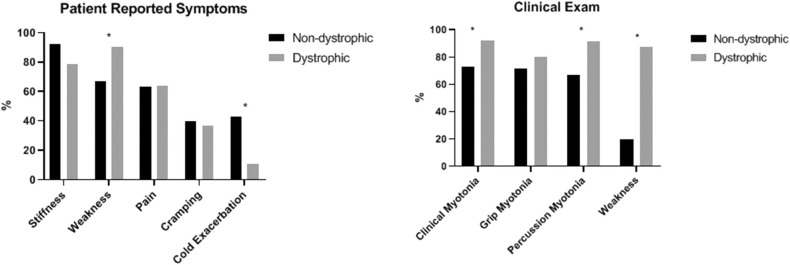
Comparison of patient reported symptoms and clinical examination between patients with non-dystrophic myotonia and dystrophic myotonia. Asterisks indicate areas with statistically significant differences. Clinical myotonia (*p* = 0.0073), percussion myotonia (*p* = 0.0051), and muscle weakness (*p* < 0.0001) were more common in the *D* cohort. Muscle weakness was more commonly reported by the *D* cohort (*P* = 0.0022) and cold exacerbation was more commonly reported by the *ND* cohort (*P* < 0.0001).

Many clinical features differentiated the *D* and *ND* cohorts. The average age of onset of symptoms was significantly younger in the *ND* cohort compared to the *D* cohort at 18.6 years (*SD* 15.1) and 29.9 years (*SD* 16.0), respectively (*p* = 0.0037). Cold exacerbation of symptoms was less commonly reported in the *D* cohort (11 vs. 42.9%, *p* < 0.0001) and muscle weakness was more commonly reported (90.7 vs. 66.7%, *p* = 0.0022). On examination, clinical myotonia (92.1 vs. 72.2%, *p* = 0.0073), percussion myotonia (91.5 vs. 66.7%, *p* = 0.0051), and muscle weakness (87.5 vs. 20%, *p* < 0.0001) were more common in the *D* cohort. Average CK levels among the two cohorts (161.9 U/L in *ND* vs. 261.1 U/L in *D, p* = 0.0708) and proportion of individuals with an abnormal CK level (28.6% *ND* vs. 51.7% *D, p* = 0.0797) appeared to differ but were not statistically significant. Patient reported symptoms and clinical examination findings between these groups are summarized in Figure 5. From a treatment standpoint, ND patients were significantly more likely to have trialed (88.1% *ND* vs. 31% *D, p* < 0.001) and to be currently taking an antimyotonia agent than were the individuals with dystrophic myotonia (61.9% *ND* vs. 14% *D, p* < 0.0001). Furthermore, *ND* patients were more likely to remain on a medication after trialing than *D* patients (70.3% *ND* vs. 45.2% *D, p* < 0.0001).

### CLCN1 vs. SCN4A

**Figure 6:**
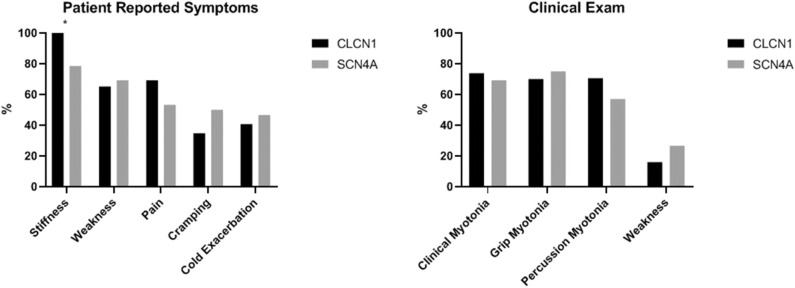
Comparison of patient reported symptoms and clinical examination between patients with variants in *CLCN1* and *SCN4A*. Asterisks indicate areas with statistically significant differences. Patients with *CLCN1* variants were more likely to report stiffness than those with *SCN4A* variants (*p* = 0.0431).

The only significant difference identified between these two cohorts was a greater proportion of individuals reporting stiffness in the *CLCN1* cohort. In our cohort, 100% (*n* = 24) of individuals with *CLCN1* variants reported stiffness compared to 78.6% (*n* = 11) of individuals with *SCN4A* variants (*p* = 0.0431). Surprisingly, weakness (65.2% *CLCN1* vs. 69.2% *SCN4A, p* = 1.0000) and pain (69.2% *CLCN1* vs. 53.3% *SCN4A, p* = 0.3357) were not significantly different between these cohorts. Patient reported symptoms and clinical examination findings between these groups are summarized in Figure 6.

## Discussion

In this retrospective study, we performed a comprehensive review of medical record data from a large group of patients with genetically confirmed dystrophic and non-dystrophic myotonic disorders. Our goals included an improved understanding of the phenotypic presentation of hereditary myotonic disorders, as well as characterization of medication use in affected persons. Utilizing this data, we are able to summarize symptom profiles and compare phenotypic features between genotypes. Additionally, we reviewed antimyotonia treatment usage, which has been understudied for this group of disorders.

Muscle stiffness is the primary clinical symptom attributed to myotonia (3). Interestingly, the prevalence of stiffness was the only feature that differed significantly among patients with non-dystrophic myotonias, with more individuals with *CLCN1* variants reporting this symptom than individuals with *SCN4A* variants. In contrast, Trivedi et al., found that 100% of both *CLCN1* and *SCN4A* patients reported stiffness (23). Although eyelid myotonia has been previously identified as a hallmark of *SCN4A*-related myotonia, we were unable to characterize its prevalence in this cohort, as it was not commonly commented on in the charts reviewed (1, 6, 7).

We found that the majority of patients with non-dystrophic myotonic disorders reported weakness and that there was no significant difference in the occurrence of these symptoms with respect to genotype. The high prevalence of reported weakness is particularly notable given that the majority (85%) of our *CLCN1* cohort had a single variant identified, and dominant CLCN1-related myotonia has not been classically associated with weakness. Patient-reported weakness in non-dystrophic myotonia has also been identified in two prospective studies; however, the proportion of individuals manifesting weakness differed by genotype. Trip et al. found that patient-reported weakness was almost twice as common in individuals with *CLCN1* variants (75%) compared to *SCN4A* variants (36.7%) while Trivedi et al. found episodic weakness to be approximately twice as common in individuals with *SCN4A* variants (76.5%) compared to individuals with *CLCN1* variants (37.5%) (22, 23). These disparate findings may reflect the different proportion of individuals with dominant versus recessive inheritance in the *CLCN1* cohorts. The majority of the Trip et al. cohort had recessive *CLCN1* variants (previously reported to be associated with a higher incidence of muscle weakness), while the cohort in the Trivedi et al. study had approximately equivalent proportions of dominant and recessive variants (22, 23). Alternatively, these variable findings could be due to differences in symptom ascertainment. Trip et al. documented the presence of “muscle weakness,” while Trivedi et al. documented “episodic weakness.” In future studies, it may be helpful to ascertain patient-reported weakness in several different ways to better characterize the spectrum of weakness and its functional impact for affected persons.

MMT data revealed that 20% of our non-dystrophic cohort had weakness that was identified via clinical examination. In a *SCN4A* cohort reported by Matthews et al., four out of seventeen (23.5%) had weakness, none with strength of <4/5 (24). Similarly, we identified clinical weakness in 26.7% of our *SCN4A* cohort. Data correlating weakness identified on MMT with *CLCN1* variants has not been published, but within our cohort was present in 16% (*n* = 4). The presence of muscle weakness on MMT is considered uncommon in individuals with non-dystrophic myotonia, despite the fact that a large proportion of these patients report weakness as a symptom (5, 6, 22, 23). Additionally, weakness that is episodic or associated with a specific trigger may be missed during standard strength testing, which could account for some of the discrepancy between patient report and clinical examination.

Pain was reported in a high proportion of all cohorts, affecting 63.7% overall. Furthermore, we found that similar proportions of patients with *CLCN1* variants and *SCN4A* variants experience pain, at 69.2 and 53.3%, respectively. Although painful myotonia is an accepted clinical feature of *SCN4A*-related myotonia, *CLCN1-*related myotonia was originally described as being painless (1). Prior studies have reported pain in 57–82% of individuals with *SCN4A* variants and in 28–53% of individuals with *CLCN1* variants (22, 23). We found that pain was reported in all DM2 patients and 59.5% of patients with DM1. Presence of pain is often considered more common in individuals with DM2, which our data supports (25, 26). Identification of pain and weakness in a high proportion of patients, particularly in those with disease not historically associated with these symptoms, is important in understanding phenotype, disease burden, and potential treatment opportunities.

Another aim of our study was to understand the treatment approaches in patients with inherited myotonic disorders. Currently, there are no medications that are approved for the treatment of myotonia, but a number of medications are used off label. In our study cohort, 47.9% of patients trialed at least one medication for myotonia and 28.2% were taking an antimyotonia medication at the time of chart review. Trivedi et al. similarly found that 60.6% of their cohort (*CLCN1, SCN4A, CNBP)* was currently taking an antimyotonia agent, but did not specify medication usage by genotype; and DM1 patients were not studied (19). In our cohort, 83% of all patients reported stiffness and 64% reported pain. This suggests that some symptomatic individuals are not being treated, and lack of medication utilization cannot be explained by lack of symptoms. Individuals with non-dystrophic myotonia were significantly more likely to have trialed and remained on an antimyotonia agent. Of those who tried an antimyotonia medication, non-dystrophic patients were also more likely to have trialed two or more medications than the dystrophic patients. Given that clinical myotonia was more common in the dystrophic cohort, and that patient-reported myotonia was similar in the dystrophic and non-dystrophic cohorts, the different rates of medication usage are evidently not due to differences in symptom prevalence. Although it is possible that the non-dystrophic cohort experienced less symptoms due higher medication use, charts were reviewed at multiple time points, including the initial patient visit, to reduce the effect of medication on these data points. Other possible explanations for this discrepancy include: non-dystrophic patients may have better therapeutic response to currently available medications, non-dystrophic patients may be more compliant in taking medications or due to the lack of systemic symptoms in non-dystrophic patients, the focus for therapy may be on their myotonia symptoms rather than symptoms in other body systems. None the less, this data suggests that there could be a gap between patients who may benefit from use of antimyotonia agents and those who are actually treated. Ideally, treatable symptoms should be ascertained and medication offered for patients who may benefit. Further study of medical treatment of myotonia, including genotype-specific treatment, dose, duration, side effects, reasons for discontinuation, and non-compliance is necessary to optimize symptom management for patients affected with these disorders.

This study has limitations associated with retrospective chart review and single center bias. These include limitations in type of data that is charted and available to review, lack of consistency in the type and depth of information recorded in the medical record, variability in number of appointments and time between appointments for patients included and the possibility that patients may be not reporting all symptoms to a physician or falsely reporting presence or absence of symptoms and/or treatment response. Consistency of type of data recorded, and format in which it was recorded, was controlled by utilization of a standardized database which aided in consistency during the data entry process.

Inherited myotonic disorders present diagnostic and treatment challenges in the clinical setting. Different types of myotonic disorders may be difficult to distinguish clinically, emphasizing the importance of comprehensive genetic testing. In patients suspected of having a non-dystrophic myotonia, the most expeditious, and cost-effective approach is panel testing including *CLCN1* and *SCN4A* sequencing. We found that the majority of patients with myotonic disorders had symptoms of pain, weakness, and stiffness. This includes individuals with non-dystrophic myotonia, a group where pain and weakness have not always been considered common symptoms. Despite this, only about half of the patients in this study were treated with antimyotonia agents. Advances in pharmacologic treatments for myotonia are needed, as there are no medications specifically approved for the indication of myotonia. Future studies should be designed to investigate relationships between specific genetic variants, clinical phenotypes, and symptom profiles, as well as response to different potential antimyotonia treatments.

## Data Availability Statement

All datasets generated for this study are included in the article/Supplementary Material.

## Ethics Statement

This project was approved by the Ohio State Biomedical Sciences IRB.

## Author Contributions

AM performed chart reviews and data entry. RS and AM created the database. RS and DK performed statistical analysis. AM, JR, SL, and WA wrote the manuscript. All authors were involved in the design of this study.

## Conflict of Interest

WA received funding from Gilead Sciences, the Neuroscience Research Institute at The Ohio State University, and has served as a paid consultant to Genentech and La Hoffmann Roche. The remaining authors declare that the research was conducted in the absence of any commercial or financial relationships that could be construed as a potential conflict of interest.
