# Supplementary material for: Genotype-Phenotype Correlations and Characterization of Medication Use in Inherited Myotonic Disorders
Source: Front Neurol. 2020 Jun 26;11:593. doi: 10.3389/fneur.2020.00593 (PMC7332828; doi:10.3389/fneur.2020.00593)
Supplement: Supplementary file 1 [file Table_1.DOCX]

**Supplementary Table 1.** Additional genetic testing performed for patients with variants of unclear significance to phenotype. This includes: 1) patients with a single variant identified which was previously associated with recessively inherited disease **(AR)**, 2) patients with a variant of uncertain significance per original report or ClinVar **(VUS)** and/or 3) patients who had variants with conflicting interpretations in ClinVar **(conflicting)**. The “conflicting” category does not include patients whose variants were conflicting between likely pathogenic and pathogenic, only for those in which one lab classified the variant a VUS or lower. Additional genetic testing reviewed included testing in *CLCN1, SCN4A, DMPK* and/or *CNBP*. All results of the additional tests listed were negative.

| Gene | c./p. | Reason for Unclear Significance | Additional Negative Genetic Testing | Testing Details (for *CLCN1* and *SCN4A*) |
| --- | --- | --- | --- | --- |
| CLCN1 | c.469delC/p.Leu157Phefs*13 | AR | DMPK, SCN4A | NGS; sequencing and del/dup; full gene |
| CLCN1 | c.501C>G/p.Phe167Leu | VUS, conflicting | DMPK, SCN4A | NGS; sequencing and del/dup; full gene |
| CLCN1 | c.592C>G/p.Leu198Val | VUS, conflicting | SCN4A | NGS; sequencing and del/dup; full gene |
| CLCN1 | c.1167-10T>C/intronic | AR | DMPK, CNBP, SCN4A | NGS; sequencing only; full gene |
| CLCN1 | c.1444G>C/p.Gly482Arg | AR | DMPK, SCN4A | NGS; sequencing and del/dup; full gene |
| CLCN1 | c.2848G>A/p.Glu950Lys | VUS | none | NGS; sequencing only; full gene |
| CLCN1 | c.568G>A/p.Gly190Arg  c.1238T>G/p.Phe413Cys | VUS, conflicting | DMPK, CNBP | NGS; sequencing only; full gene |
| CLCN1 | c.979G>A/p.Val327Ile  c.1262G>T/p.Arg421Leu | VUS | none | NGS; sequencing only; full gene |
| SCN4A | c.4372G>T/p.Val458Phe | VUS | none | NGS; sequencing and del/dup; full gene |
| SCN4A | c.4386C>G/p.Ile1462Met | VUS | none | NGS; sequencing only; full gene |
| SCN4A | c.5126A>G/p.Asn1709Ser | VUS | DMPK, CNBP, CLCN1 | NGS; sequencing and del/dup; full gene |

Abbreviations: NGS = next generation sequencing; del/dup = deletion and duplication analysis; full gene = entire gene was analyzed during testing; VUS = variant of uncertain significance
